# The therapeutic effect of the BRD4-degrading PROTAC A1874 in human colon cancer cells

**DOI:** 10.1038/s41419-020-03015-6

**Published:** 2020-09-25

**Authors:** An-cheng Qin, Hua Jin, Yu Song, Yun Gao, Yi-Fan Chen, Li-na Zhou, Shu-sheng Wang, Xing-sheng Lu

**Affiliations:** 1 Department of Hepatobiliary Surgery, Suzhou Municipal Hospital Affiliated to Nanjing Medical University, Suzhou, China; 2 The Child Health Care Department, Suzhou Ninth People’s Hospital, Suzhou, China; 3 Department of Oncology, The Affiliated Zhangjiagang Hospital of Soochow University, Suzhou, China; 4 Trauma center, Affiliated Hospital of Yangzhou University, Yangzhou, China; 5 Department of Radiotherapy and Oncology, Affiliated Kunshan Hospital of Jiangsu University, Kunshan, China; 6 Department of General Surgery, The Affiliated Zhangjiagang Hospital of Soochow University, Suzhou, China

**Keywords:** Apoptosis, Oncogenes

## Abstract

A1874 is a novel BRD4-degrading proteolysis targeting chimera (PROTAC). In primary colon cancer cells and established HCT116 cells, A1874 potently inhibited cell viability, proliferation, cell cycle progression, as well as cell migration and invasion. The BRD4-degrading PROTAC was able to induce caspase and apoptosis activation in colon cancer cells. Furthermore, A1874-induced degradation of BRD4 protein and downregulated BRD-dependent genes (*c-Myc*, *Bcl-2*, and *cyclin D1*) in colon cancer cells. Significantly, A1874-induced anti-colon cancer cell activity was more potent than the known BRD4 inhibitors (JQ1, CPI203, and I-BET151). In BRD4-knockout colon cancer cells A1874 remained cytotoxic, indicating the existence of BRD4-independent mechanisms. In addition to BRD4 degradation, A1874 cytotoxicity in colon cancer cells was also associated with p53 protein stabilization and reactive oxygen species production. Importantly, the antioxidant N-acetyl-cysteine and the p53 inhibitor pifithrin-α attenuated A1874-induced cell death and apoptosis in colon cancer cells. In vivo, A1874 oral administration potently inhibited colon cancer xenograft growth in severe combined immuno-deficient mice. BRD4 degradation and p53 protein elevation, as well as apoptosis induction and oxidative stress were detected in A1874-treated colon cancer tissues. Together, A1874 inhibits colon cancer cell growth through both BRD4-dependent and -independent mechanisms.

## Introduction

Colon cancer has become a common malignancy and a global health issue^1,2^, causing significant cancer-related human mortalities each year^3,4^. Current clinical treatments, including chemotherapy, surgery, radiation, and/or molecularly targeted therapies^1,5,6^, along with the advanced early screen and diagnosis techniques, have significantly improved the prognosis and five-year overall survival of colon cancer patients^1,5,6^. Yet the prognosis for the advanced, metastatic and recurrent colon cancer patients remains poor^1^. There is an urgent need to further explore the underlying pathological mechanisms of colon cancer development and progression^1,5,6^.

Bromodomain-containing protein 4 (BRD4) is an extensively studied BET (bromodomain and extra-terminal domain) family protein, that provides a promising therapeutic target for colon cancer^7^. BRD4 directly binds to acetylated histones and plays an essential role in regulating epigenetic processes^8–10^. In the process of mitosis, BRD4 is required for chromatin structure formation in the daughter cells^9,11^. Furthermore, BRD4 is important for transcription elongation and expression of key oncogenic genes including Bcl-2 and c-Myc. BRD4 associates with positive transcription elongation factor b (P-TEFb) to phosphorylate RNA polymerase II in proliferating cells^9,11^. Recent studies have proposed BRD4 as a novel oncogene as it is overexpressed in colon cancer^12^ and many other malignancies^8,11^.

Multiple small-molecule inhibitors of BRD4 have been developed, showing promising anticancer results in experimental and clinical cancer studies^8,11,13^. However, BRD4 inhibition results in feedback elevation of BRD4 protein in human cancer cells, leading to weak anti-proliferative activity and less apoptosis induction^8,11,13^. A recent study identified A1874 as a BRD4-targeting mouse double minute 2 homolog (MDM2)-based proteolysis targeting chimera (PROTAC)^14^.

Unlike the BRD4 inhibitors, A1874 can lead to a robust and sustained BRD4 protein degradation through ubiquitin system^14^, causing profound inhibition of BRD4-dependent cancers^14^. In this study, we tested the potential anticancer activity and underlying signaling mechanisms of A1874 against human colon cancer cells.

## Materials and methods

### Chemicals, reagents, and antibodies

A1874 was obtained from Hanxiang BioTech (Shanghai, China). Puromycin, JQ1, CPI203, I-BET151, N-acetyl-cysteine (NAC) and pifithrin-α, polybrene and CCK-8 were purchased from Sigma-Aldrich (St. Louis, MO). All cell culture reagents were provided by Hyclone Co. (Logan, UT). Antibodies for c-Myc (#9402), Cyclin D1 (#2922), BRD4 (#13440), Bcl-2 (#15707), Erk1/2 (#9102), p53 (#9282), cleaved-caspase-3 (#9664), cleaved-caspase-9 (#20750), cleaved-poly (ADP-ribose) polymerase (PARP) (#5625), Bcl-2 (#15707) and β-tubulin (#15115) were purchased from Cell Signaling Tech (Beverly, MA). Caspase inhibitors, z-VAD-fmk and z-DEVD-fmk, were provided by Thermo-Fisher (Shanghai, China). Lipofectamine 2000, TUNEL (terminal deoxynucleotidyl transferase dUTP nick end labeling), Annexin V and propidium iodide (PI) were purchased from Thermo-Fisher Invitrogen (Carlsbad, CA). The BrdU ELISA kit was provided by Roche Diagnostics (Basel, Switzerland).

### Cell culture

The primary human colon cancer cells from four written-informed consent primary colon cancer patients, pCan1/2/3/4, and primary human colon epithelial cells from two healthy donors, pEpi1/2, were provided by Dr. Lu at Nanjing Medical University^15–17^. The primary human cells were cultured in the described medium^15,18^. The established HCT116 colon cancer cells were also provided by Dr. Lu^15–17^. All the established and primary cells were subjected to routine mycoplasma and microbial contamination examination every 2–3 months. To confirm the genotype of the cells, STR (short tandem repeat) profiling, population doubling time, and cell morphology were regularly checked. The protocols of this study were approved by Ethic Committee of Nanjing Medical University, according to Declaration of Helsinki.

### Cell viability assay

The viable colon cancer cells or colon epithelial cells were seeded into 96-well plates at 5 × 10^3^ cells per well. CCK-8 kit was utilized to determine cell viability, with CCK-8 optical densities (ODs) recorded at 550 nm.

### Colony formation

Colon cancer cells with the applied A1874 treatment were re-suspended in 1 mL of DMEM with 0.5% agar (Sigma). Cells were then added onto the pre-solidified 10-cm cell culture dish. Medium was renewed every 2 days for a total of five rounds. Afterwards, the remaining cell colonies were stained and manually counted.

### BrdU ELISA

Cells were seeded into 96-well plates at 5 × 10^3^ cells per well. With the applied A1874 treatment, BrdU incorporation was tested by a BrdU ELISA kit according to the attached protocol. BrdU ELISA absorbance was recorded at 405 nm.

### EdU staining

The viable colon cancer cells were seeded into 12-well plates (8 × 10^4^ cells per well). With the applied A1874 treatment, EdU (5-ethynyl-20-deoxyuridine) staining assay was performed as described elsewhere^19,20^. EdU percentages (EdU vs. DAPI, %) of 1000 cells per treatment in five random views (under a fluorescence microscope at 1× 100 magnification) were recorded.

### Cell migration and invasion assays

Colon cancer cells (3 × 10^4^ cells per chamber, in serum-free medium) were seeded onto the “Transwell” chambers: (8-μm pore size, Corning Costar, Shanghai, China). Complete medium containing 10% FBS was added to the lower chambers^21^. After incubation for 24 h, the migrated cells were fixed and stained. For cell invasion assays, chambers were always coated with Matrigel (Sigma)^22^. Average number of migrated/invaded cells in five random views per treatment were recorded.

### Cell cycle FACS

The primary human colon cancer cells were seeded into six-well plate at 2 × 10^5^ cells per well. With the applied A1874 treatment, cells were washed, fixed, and incubated with DNase-free RNase and PI. Cell cycle distribution was recorded by using a FACSCalilur machine (BD Biosciences, Shanghai, China).

### Caspase activity

The viable colon cancer cells were seeded into six-well plates (at 2 × 10^5^ cells per well). Following treatment, 30 μg of cytosolic extract lysates (per treatment) were added to the caspase assay buffer^23^ together with the applied 7-amido-4-(trifluoromethyl)-coumarin (AFC)-conjugated caspase-3/-8/-9 substrate^23^. The AFC fluorescence intensity was tested under a Fluoroskan machine^23^.

### Mitochondrial depolarization

JC-1 dye can aggregate into mitochondria and form green monomers in the apoptotic cells with mitochondrial depolarization^24^. The viable colon cancer cells were seeded into six-well plates (at 2 × 10^5^ cells per well). Following treatment, cells were incubated with JC-1 (5.0 μg/mL). JC-1 intensity was tested immediately under a fluorescence spectrofluorometer at 488 nm. The representative merged JC-1 images were presented as well.

### Annexin V-PI-FACS

The primary human colon cancer cells were seeded into six-well plate at 2 × 10^5^cells per well. Forty-eight hours after the applied A1874 treatment, cells were harvested, washed, and incubated with Annexin V and PI (10 μg/mL each). Afterwards, cells were gated under a FACSCalibur machine (BD Biosciences). The percentage of Annexin V-positive cells was always recorded.

### TUNEL staining

The viable colon cancer cells or colon epithelial cells were seeded into 12-well plates (8 × 10^4^ cells per well). Forty-eight hours after the applied A1874 treatment, cells were incubated with both TUNEL and DAPI dyes. The nuclear TUNEL percentage (TUNEL vs. DAPI, %) of 1000 cells per treatment in five random views (under a fluorescence microscope at 1× 100 magnification) was recorded.

### Trypan blue assay

Colon cancer cells were seeded into 12-well plates (8 × 10^4^ cells per well). Seventy-two hours after the applied A1874 treatment, Trypan blue dye was added. Its ratio was recorded by using an automatic cell counter.

### Western blotting

Colon cancer cells were seeded into six-well plate at 2 × 10^5^cells per well. After the applied A1874 treatment, cells were incubated with the described lysis buffer^25^. Total protein lysates (30 μg per treatment in each lane) were analyzed. Western blotting protocols were described previously^26^. Data quantification was done through the ImageJ software (NIH).

### Quantitative real-time PCR (qPCR)

Colon cancer cells were seeded into six-well plate at 2 × 10^5^ cells per well. Following the applied treatment, TRIzol reagents were utilized to extract total RNA, and the latter was converted into complementary DNA (cDNA). qPCR was carried out using a SYBR Premix Ex Taq™ kit (TaKaRa, Tokyo, Japan) under the ABI Prism 7500 Fast Real-Time PCR system. *GAPDH* was always used as the reference gene and the internal control. Quantification was performed with the 2^−ΔΔCt^ method. The RNA primer sequences employed in this study were from Dr. Zhu at Soochow University^27^.

### Reactive oxygen species (ROS) assay

Colon cancer cells were seeded into six-well plate at 2 × 10^5^ cells per well. After the applied A1874 treatment, cells were stained with CellROX dye (Beyotime, Wuxi, China) and thereafter tested via a fluorescence microscopy.

### GSH/GSSG ratio

Reduced glutathione (GSH) is a vital ROS scavenger in human cells. Its ratio with the oxidized disulfide form glutathione (GSSG) was tested as a quantitative indicator of oxidative stress intensity^28^. Colon cancer cells were seeded into six-well plate at 2 × 10^5^ cells per well. With the applied A1874 treatment, cells were lysed. The GSH/GSSG ratio was measured using a GSH/GSSG assay kit (Beyotime). GSH/GSSG ratio in human tissues was tested similarly.

### Assaying DNA breaks

The viable colon cancer cells were seeded into 96-well plates at 5 × 10^3^ cells per well. Following the applied A1874 treatment, a single strand DNA (ssDNA) ELISA kit (Roche, Shanghai, China) was utilized to test DNA breaks. The ssDNA ELISA absorbance was tested by 405 nm.

### Exogenous BRD4 overexpression

The pSUPER-puro-GFP expression vector, containing the mutant BRD4 at the MDM2 binding sites, was provided by Dr. Zhao at Soochow University^29^. It was transfected to HEK-293 cells together with viral packaging proteins (VSVG and Hit-60) (provided by Dr. Zhao^29^) to generate BRD4-expressinglentivirus. Virus was then enriched, filtered and added to cultured colon cancer cells (in polybrene-containing complete medium), and stable cells selected by puromycin. Exogenous BRD4 overexpression was verified by Western blotting.

### BRD4 knockout

A CRISPR/Cas9-BRD4-knockout (KO) plasmid (with puromycin selection gene, from Dr. Zhao at Soochow University^29^) was transfected into primary colon cancer cells via a Lipofectamine 2000 (Thermo-Fisher Invitrogen) protocol. Cells were distributed to 96-well plates to establish single cells and were subjected to BRD4-KO screening (qPCR). Stable cells were further selected by puromycin for 4–5 passages. BRD4 KO in the stable cells was always verified by Western blotting.

### Tumor xenografts

The severe combined immuno-deficient (SCID) mice (5–6 week old, 18–19 g weight, all female) were purchased from the Animal Facility of Soochow University (Suzhou, China). The primary pCan1 colon cancer cells (8 × 10^6^ cells per mouse) were subcutaneously (*s.c*.) injected to the flanks of SCID mice. Within three weeks the tumors reached the average volume of 100 mm^3^. Tumor-bearing mice were then randomly assigned into two groups (nine mice per group/*n* = 9). Mice were then treated with A1874 or the vehicle control. Tumor volumes were recorded using the described formula^30^. Estimated daily tumor growth (in mm^3^ per day) was calculated as described^16^. All animal studies were in accordance with regulations of the Institutional Animal Care and Use Committee and Ethics Committee of Nanjing Medical University (Nanjing, China).

### Statistical analysis

The investigators were blinded to the group allocation during all experiments. In vitro experiments were repeated at least three times. Data were presented as mean ± standard deviation (SD). Statistics analyses were carried out through one-way ANOVA with the Scheffe’ and Tukey Test (SPSS 23.0, SPSS, Chicago, IL). To determine significance between two treatment groups, the unpaired t test was used (Excel 2007). Significance was determined as *P* < 0.05. All the protocols of this study were approved by Ethics Committee of Nanjing Medical University.

## Results

### A1874 inhibits colon cancer cell growth, proliferation, cell cycle progression, migration, and invasion

**Fig. 1:**
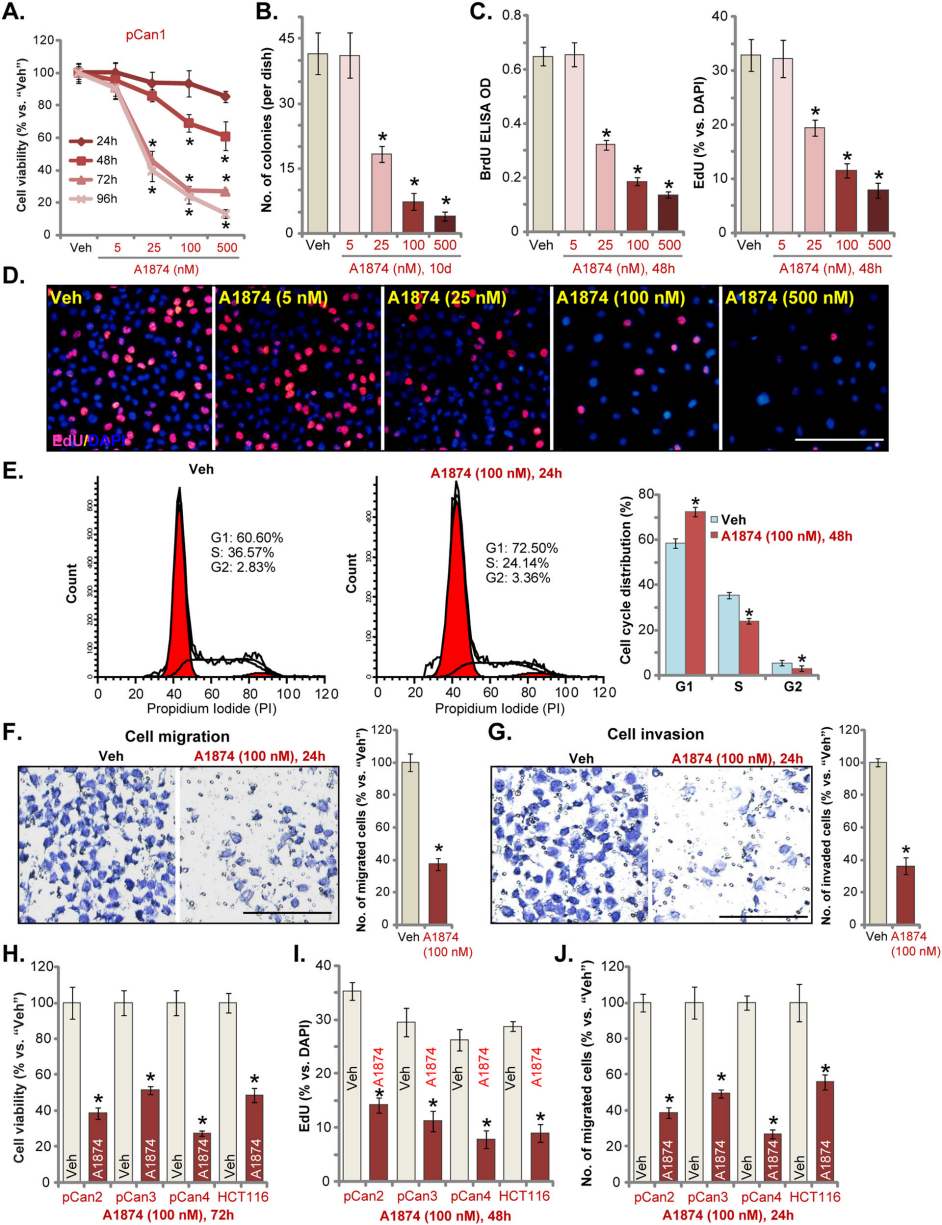
A1874 inhibits colon cancer cell growth, proliferation, cell cycle progression, migration and invasion. The primary human colon cancer cells, pCan1/2/3/4 (derived from different colon cancer patients) (**a**–**j**) or established HCT116 cells (**h**–**j**) were treated with applied concentration of A1874 (5–500 nM) or the vehicle control (“Veh,” 0.2% of DMSO). Cells were further cultured in complete medium for applied time periods, then cell viability (CCK-8 OD, **a** and **h**), colony formation (**b**) and cell proliferation (BrdU ELISA OD and nuclear EdU incorporation ratio, **c**, **d** and **i**) as well as cell cycle progression (PI-FACS, **e**), cell migration (**f**, **j**) and invasion (**g**) were tested by the listed assays. Data were presented as mean ± standard deviation (SD, *n* = 5). * *P* < 0.05 vs. “Veh” cells. Experiments in this figure were repeated three times, and similar results were obtained. Bar = 100 μm (**d**, **f**, **g**).

To examine the anti-proliferative activity of A1874, primary human colon cancer cells, pCan1, were cultured in FBS-containing complete medium and treated with increasing concentrations (5–500 nM) of A1874. A CCK-8 assay was carried out to test cell viability. As shown (Fig. 1a), A1874 decreased cell viability in pCan1 cells in a concentration-dependent manner. There was a significant reduction in viability following treatment with 25–500 nM of A1874 (Fig. 1a), whereas the lower concentration (5 nM) was ineffective (Fig. 1a). The BRD4-degrading PROTAC displayed a time-dependent response in inhibiting pCan1 cell viability (Fig. 1b). A1874 (25–500 nM) required at least 48 h to exert significant anti-survival activity (Fig. 1a). Furthermore, a colony formation assay (Fig. 1c) demonstrated that A1874 (25–500 nM) potently decreased the number of viable pCan1 cell colonies.

Additional studies showed that A1874 concentration-dependently suppressed BrdU incorporation in pCan1 cells (Fig. 1c). The percentage of cell nuclei with positive EdU staining was robustly decreased with A1874 (25–500 nM, 48 h) treatment (Fig. 1d). These results confirm the anti-proliferative activity of the BRD4-degrading PROTAC in pCan1 primary colon cancer cells. As the titration experimental results in Fig. 1a–d showed that 100 nM of A1874 efficiently inhibited pCan1 cell viability and proliferation, this concentration was selected for the additional studies.

By applying the PI-FACS assay, we found that A1874 (100 nM) resulted in cell cycle arrest by increasing the accumulation of pCan1 cells in G1-phase while reducing S- and G2/M-phase cells. Therefore, A1874-induced G1-S arrest (Fig. 1e). Examining the potential effect of A1874 on cancer cell migration, “Transwell” assay results, Fig. 1f, demonstrated that A1874 (100 nM) inhibited pCan1 cell migration in vitro. Furthermore, pCan1 cell invasion, tested by “Matrigel Transwell” assay, was suppressed by A1874 (Fig. 1g).

We also examined the potential activity of A1874 in other colon cancer cells. Primary colon cancer cells derived from three patients, pCan2/3/4, as well as established HCT116 cells, were tested. In these colon cancer cells, we found that A1874 (100 nM) potently inhibited cell viability, proliferation and migration, tested by CCK-8 (Fig. 1h), nuclear EdU incorporation (Fig. 1i) and “Transwell” (Fig. 1j) assays, respectively. Together, these studies demonstrate that A1874 potently inhibits colon cancer cell growth, proliferation, cell cycle progression, migration, and invasion.

### A1874 induces apoptosis activation in colon cancer cells

**Fig. 2:**
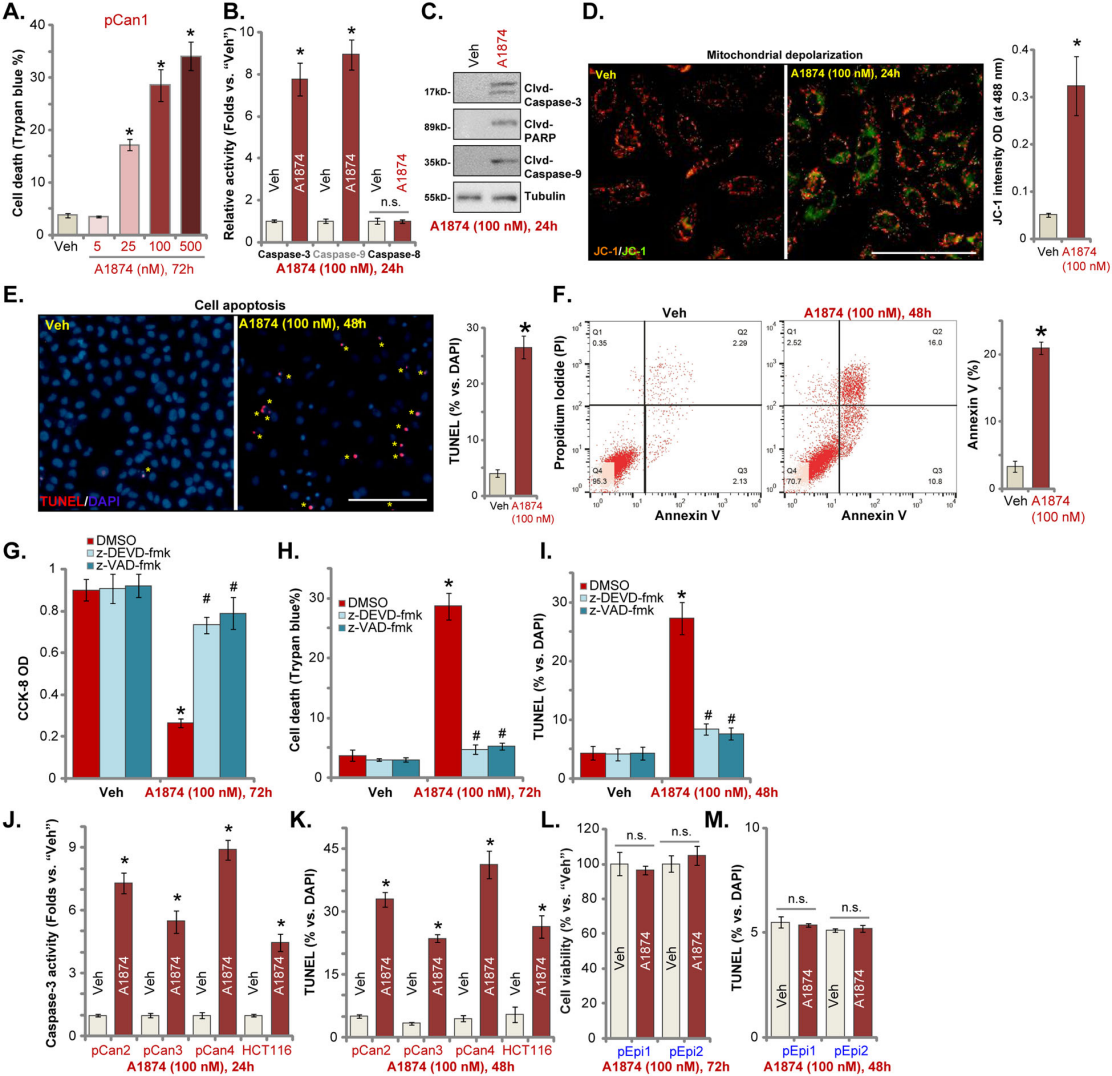
A1874 induces apoptosis activation in colon cancer cells. The primary human colon cancer cells, pCan1/2/3/4 (derived from different patients) (**a–f, j**, **k**), HCT116 cells (**j**, **k**), or primary colon epithelial cells (“pEpi1/2”) (**l, m**) were treated with applied concentration of A1874 (5–500 nM) or the vehicle control (“Veh”, 0.2% of DMSO). Cells were further cultured in complete medium for applied time periods. Then cell death (Trypan blue-positive cell ratio, **a**), caspase activation (**b**, **c**, **j**), mitochondrial depolarization (JC-1 green monomers intensity, **d**), and cell apoptosis (nuclear TUNEL staining and Annexin V FACS assays, **e**, **f**, **k**, **m**) were tested, with cell viability in epithelial cells tested by CCK-8 assay (**l**). The pCan1 primary colon cancer cells were pretreated with 50 μM of the caspase-3 inhibitor z-DEVD-fmk or the pan caspase inhibitor z-VAD-fmk for 30 min, followed by A1874 (100 nM) stimulation and cultured for 72 h; Then cell viability, death and apoptosis were tested by CCK-8 (**g**), Trypan blue staining (**h**) and nuclear TUNEL staining (**i**) assays, respectively. TUNEL-positive nuclei were marked by the yellow stars (**e**). Data were presented as mean ± standard deviation (SD, *n* = 5). *P < 0.05 vs. “Veh” cells. ^#^*P* < 0.05 vs. A1874 (100 nM) only treatment (**g**–i). “n.s.” stands for no statistic difference (**l**, **m**). Experiments in this figure were repeated three times, and similar results were obtained. Bar = 100 μm (**d**, **e**).

As proliferation inhibition and cell cycle arrest can induce cell apoptosis in colon cancer cells^15,31,32^, we tested whether A1874 can provoke cell death and apoptosis. Trypan blue staining assay results, Fig. 2a, confirmed that A1874 dose-dependently induced pCan1 colon cancer cell death (increased Trypan blue staining). A1874 (100 nM, 24 h) significantly increased caspase-3 and caspase-9 activity in pCan1 cells (Fig. 2b), leaving caspase-8 activity unaffected (Fig. 2b). Furthermore, cleavage of caspase-3, caspase-9, and poly ADP-ribose polymerase (PARP) was detected in A1874-treated pCan1 cells (Fig. 2c). A1874 also induced mitochondrial depolarization and caused JC-1 green monomers accumulation in the mitochondria of pCan1 cells (Fig. 2d). These results confirm the activation of mitochondrial apoptosis cascade^33–36^ following A1874 treatment (Fig. 2b–d). Further, A1874 (100 nM) induced apoptosis activation in pCan1 cells, causing increased ratios of TUNEL-positive nuclei (Fig. 2e) and Annexin V-positive cells (Fig. 2f).

Significantly, the caspase-3 inhibitor z-DEVD-fmk and the pan caspase inhibitor z-VAD-fmk largely inhibited A1874 (100 nM)-induced decreased viability (CCK-8 OD) (Fig. 2g), cell death (Trypan blue staining assay, Fig. 2h), and apoptosis activation (nuclear TUNEL staining assay, Fig. 2i). Therefore, apoptosis activation appears to be the primary mechanism of A1874-induced cytotoxicity against pCan1 cells.

In other primary colon cancer cells (pCan2/3/4) and established HCT116 cells, A1874 (100 nM) treatment robustly increased caspase-3 activity (Fig. 2j) and TUNEL-positive nuclear ratio (Fig. 2k), indicating apoptosis activation. However, in the primary colon epithelial cells (“pEpi1/2,” from two donors^18^), A1874 (100 nM) treatment failed to decrease cell viability (Fig. 2l) and induce apoptosis activation (Fig. 2m), indicating a cancer cell-specific effect by the BRD4-degrading PROTAC.

### A1874-induced anti-colon cancer cell activity is not solely dependent on BRD4 protein degradation

**Fig. 3:**
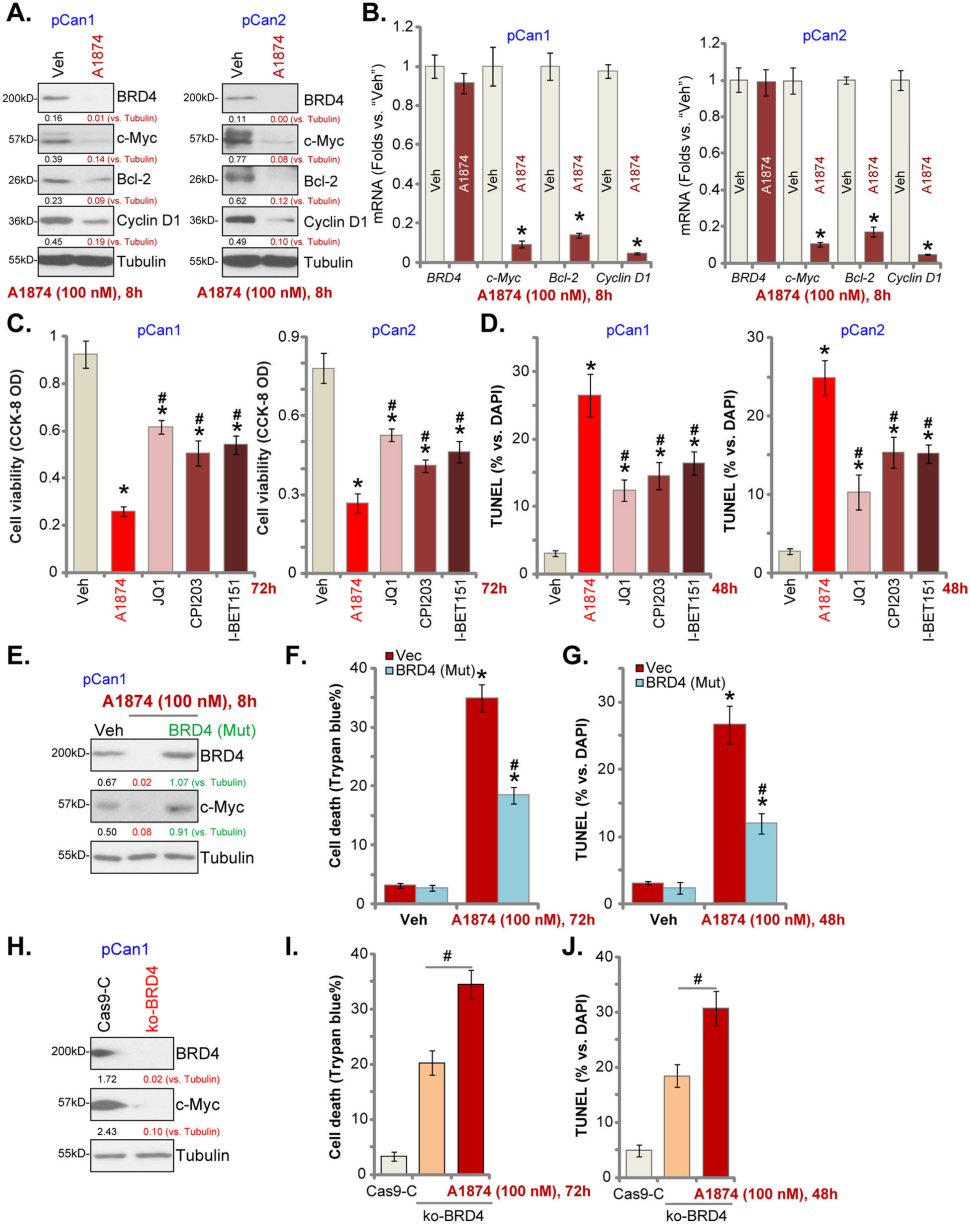
A1874-induced anti-colon cancer cell activity is not solely dependent on BRD4 protein degradation. The primary human colon cancer cells, pCan1 and pCan2, were treated with A1874 (100 nM) or the vehicle control (“Veh”, 0.2% of DMSO).Cells were further cultured in complete medium for applied time periods, and then expression of listed proteins (**a**) and mRNAs (**b**) were shown. The primary human colon cancer cells, pCan1 and pCan2, were treated with A1874 (100 nM), JQ1 (500 nM), I-BET726 (200 nM), CPI203 (500 nM) or the vehicle control (“Veh,” 0.2% of DMSO) and further cultured in complete medium for applied time periods, and then cell viability and apoptosis were tested by CCK-8 (**c**) and nuclear TUNEL staining (**d**) assays, respectively. Stable pCan1 cells with the mutant BRD4 expression construct [“BRD4 (Mut)”] or the empty vector (“Vec”) were treated with or without A1874 (100 nM). Cells were then further cultured in complete medium for applied time periods, and expression of listed proteins was shown (**e**); Cell death and apoptosis were tested by Trypan blue staining (**f**) and nuclear TUNEL staining (**g**) assays, respectively. The stable pCan1 cells with CRISPR/Cas9-BRD4-KO-GFP construct (“ko-BRD4” cells) were treated with or without A1874 (100 nM). The control cells with CRISPR/Cas9 empty vector (“Cas9-C”) were left untreated; Cells were further cultured in complete medium for applied time periods, and then expression of listed proteins was shown (**h**); Cell death and apoptosis were tested by Trypan blue staining (**i**) and nuclear TUNEL staining (**j**) assays, respectively. Expression of listed proteins was quantified and normalized to the loading control (**a**, **e**, **h**). Data were presented as mean ± standard deviation (SD, *n* = 5). *P < 0.05 vs. “Veh” cells. ^#^*P* < 0.05 vs. A1874 treatment (**c**, **d**). ^#^*P* < 0.05 vs. “Vec” cells (**f**, **g**). ^#^*P* < 0.05 (**I** and **J**). Experiments in this figure were repeated three times, and similar results were obtained.

Because A1874 is a novel BRD4-degrading PROTAC^14^, we tested its effect on BRD4 signaling in colon cancer cells. In primary human colon cancer cells, pCan1 and pCan2, A1874 (100 nM) treatment led to robust degradation of BRD4 protein (Fig. 3a) without affecting *BRD4* mRNA expression (Fig. 3b). Furthermore, mRNA and protein expression of BRD4-dependent genes, including *c-Myc*, *Bcl-2*, and *cyclin D1*, was significantly decreased in A1874-treated colon cancer cells (Fig. 3a, b). We then compared the anti-colon cancer cell activity of A1874 with other known BRD4-BET inhibitors, including JQ1^37,38^, CPI203^29,39^, and I-BET726^40–42^. In pCan1 cells and pCan2 cells, A1874-induced viability (CCK-8 OD) reduction (Fig. 3c), and cell apoptosis (nuclear TUNEL staining assay, Fig. 3d) were significantly more potent than JQ1, CPI203, and I-BET726. These known inhibitors were utilized at even higher concentrations than A1874. These results suggest that A1874-induced anti-colon cancer cell activity might not be solely dependent on BRD4 protein degradation.

To explore whether A1874 acts solely to promote BRD4 protein degradation, a mutant BRD4 expression construct [“BRD4 (Mut)”], with a mutation at the MDM2’s binding site^14^, was stably transduced into pCan1 cells. Western blotting assay results, Fig. 3e, demonstrated that BRD4 and c-Myc protein expression was restored by the BRD4 (Mut) construct even after A1874 treatment. However, the BRD4 (Mut) only partially inhibited A1874 (100 nM)-induced cell death (Trypan blue ratio increase, Fig. 3f) and apoptosis (nuclear TUNEL staining assay, Fig. 3g). These studies support the existence of BRD4-independent mechanisms responsible for A1874-induced cytotoxicity in colon cancer cells.

To further support our hypothesis, we found that CRISPR/Cas9-induced BRD4 KO resulted in c-Myc downregulation (Fig. 3h), causing pCan1 cell death (Fig. 3i) and apoptosis (Fig. 3j). In BRD4-KO cells, A1874 (100 nM) was able to induce further cell death (Fig. 3i) and apoptosis (Fig. 3j). Thus, in addition to BRD4 protein degradation, parallel cell death mechanisms are also responsible for A1874-induced anti-colon cancer cell activity.

### A1874 induces p53 protein stabilization and oxidative injury in colon cancer cells

**Fig. 4:**
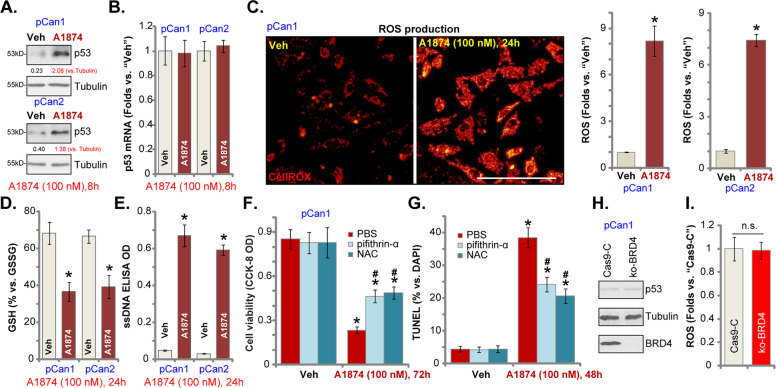
A1874 induces p53 protein stabilization and oxidative injury in colon cancer cells. The primary human colon cancer cells, pCan1 and pCan2, were treated with A1874 (100 nM) or the vehicle control (“Veh”, 0.2% of DMSO). Cells were further cultured in complete medium for applied time periods, and then expressions of p53 protein (**a**) and mRNA (**b**) were shown; The CellROX intensity (**c**), the GSH/GSSG ratio (**d**) and the single strand DNA (ssDNA) contents (**e**) were tested as well. The pCan1 cells were pretreated for 1 h with the antioxidant N-acetyl-cysteine (NAC, 400 μM) or the p53 inhibitor pifithrin-α (10 μM), followed by A1874 (100 nM) stimulation for another 48–72 h.Then cell viability was tested by CCK-8 assay (**f**), with cell apoptosis examined by nuclear TUNEL staining assay (**g**). Stable pCan1 cells with CRISPR/Cas9-BRD4-KO-GFP construct (“ko-BRD4” cells) or control cells with CRISPR/Cas9 empty vector (“Cas9-C”) were cultured for 24 h, and then expression of listed proteins (**h**) and ROS contents (CellROX intensity, **i**) were tested. Expression of listed proteins was quantified and normalized to the loading control (**a**). Data were presented as mean ± standard deviation (SD, *n* = 5). *P < 0.05 vs. “Veh” cells. ^#^*P* < 0.05 vs. A1874 treatment (**f**, **g**). Experiments in this figure were repeated three times, and similar results were obtained. Bar = 100 μm (**c**). “n.s.” stands for no statistic difference (**i**).

Possible A1874-induced parallel cell death mechanisms include p53 protein stabilization and oxidative injury. A1874 is a novel BRD4-targeting MDM2-based PROTAC, reported to stabilize p53 protein^14^. In the primary colon cancer cells, pCan1 and pCan2, treatment with A1874 (100 nM, 8 h) induced robust p53 protein elevation (Fig. 4a), whereas expression of *p53* mRNA was unchanged (Fig. 4b). Interestingly, A1874-induced significant oxidative injury in colon cancer cells, increasing CellROX fluorescence intensity^43^ in pCan1 and pCan2 cells (Fig. 4c). A1874-induced oxidative stress in colon cancer cells was also indicated by the GSH/GSSG ratio reduction (Fig. 4d) and ssDNA accumulation (Fig. 4e, DNA breaks).

To study whether p53 protein stabilization and oxidative injury participate in A1874-induced anti-colon cancer cell activity, the antioxidant NAC and the p53 inhibitor pifithrin-α^44,45^ were applied. As shown, A1874 (100 nM)-induced viability (CCK-8 OD) reduction was inhibited by NAC and pifithrin-α in pCan1 cells (Fig. 4f). Furthermore, NAC and pifithrin-α mitigated A1874-induced pCan1 cell apoptosis (nuclear TUNEL staining assay, Fig. 4g). Significantly, CRISPR/Cas9-induced BRD4 KO (see Fig. 3) failed to promote p53 protein upregulation (Fig. 4h) and ROS production (CellROX intensity, Fig. 4i) in pCan1 cells. These results demonstrate that p53 stabilization and oxidative injury act independently of BRD4 protein degradation to participate in A1874-induced anti-colon cancer cell activity.

### A1874 oral administration inhibits colon cancer xenograft growth in SCID mice

**Fig. 5:**
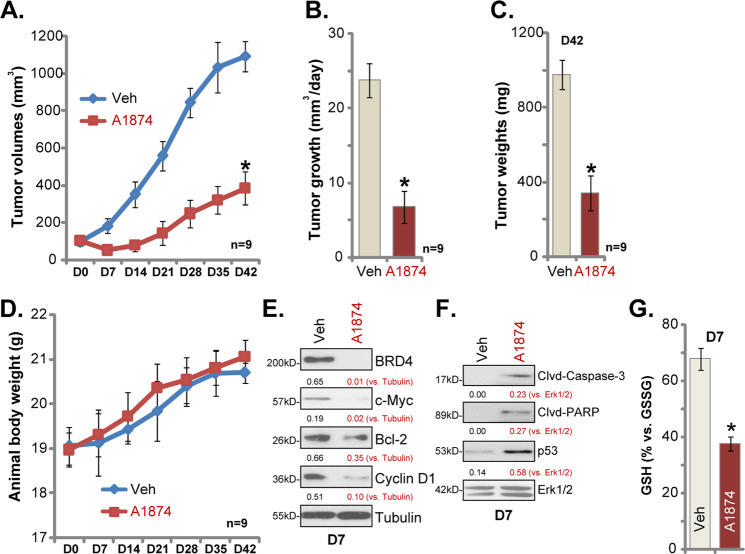
A1874 oral administration inhibits colon cancer xenograft growth in SCID mice. The SCID mice bearing pCan1 colon cancer xenografts were treated with A1874 (20 mg/kg body weight, oral administration, daily for 21 days) or the vehicle control (“Veh”); Tumor volumes (**a**) and mice body weights (**d**) were recorded weekly. The estimated daily tumor growth was calculated by using the described formula (**b**). At Day-42/D42, tumors of the two groups were isolated and weighted (**c**). At treatment Day7 (D7), one tumor of each group was isolated, and tumors were homogenized. Expression of the listed proteins in tumor tissue lysates was tested (**e**, **f**). The GSH/GSSG ratio in tumor tissue lysates was examined (**g**). Expression of listed proteins was quantified and normalized to the loading control (**e**, **f**). Data were presented as mean ± standard deviation (SD). *P < 0.05 vs. “Veh” group.

In order to study the potential anticancer activity of A1874 in vivo, pCan1 colon cancer cells were *s.c*. injected into the flanks of SCID mice. Within three weeks, colon cancer xenografts were established with tumor volumes close to 100 mm^3^ (Day-0/D0). By recording tumor growth curve, we demonstrated that A1874 oral administration (20 mg/kg, daily, 21 days) potently inhibited colon cancer xenograft growth in SCID mice (Fig. 5a). Calculating the estimated daily tumor growth, using the formula: (Tumor volume at D42—Tumor volume at D0)/42 (days), we found that colon cancer xenograft growth was largely inhibited in A1874-treated mice (Fig. 5b). Tumors from the two groups were isolated and weighted individually at experimental Day-42 (D42). Xenograft tumors with A1874 administration were significantly lighter than those of vehicle control mice (Fig. 5c). Mouse body weights were not significantly different between A1874-treated and vehicle control mice (Fig. 5d), and no noticeable toxicity was observed in the mice. These results show that oral administration of A1874 is able to inhibit colon cancer xenograft growth in SCID mice.

At treatment D7, one tumor from each group was isolated, and tumors were homogenized. Western blotting analyses showed that protein expression of BRD4, c-Myc, Bcl-2 and cyclin D1, were significantly decreased in A1874-treated tumor tissues (Fig. 5e), where caspase-3 and PARP cleavage was detected (Fig. 5f). Furthermore, p53 protein elevation was observed in xenograft tissues with A1874 administration (Fig. 5f). Additionally, the GSH/GSSG ratio was decreased in A1874-treated xenograft tissue lysates, indicating oxidative stress (Fig. 5g). Therefore, in line with in vitro findings, A1874 was able to induce BRD4 protein degradation, apoptosis activation, p53 elevation and oxidative stress in colon cancer xenografts.

## Discussion

BRD4, a gene that is overexpressed in different human cancers, is associated with carcinogenesis, tumorigenesis, and progression of human malignancies. It is emerging as a promising therapeutic target^8,46,47^. The development and optimization of BRD4 small-molecule inhibitors as novel cancer therapeutics are currently a major focus of cancer research^8,46,47^. BRD4 binds directly to the acetylated histones to promote transcription and expression of multiple oncogenic genes, including c-Myc and several others^8,46,47^. BRD4 also acts as an associated factor of P-TEFb, stimulating RNA polymerase II-dependent transcription and cell cycle progression^10,47,48^.

BRD4 overexpression is detected in colorectal cancer (CRC)^12^. Hu et al., showed that MS417, a BRD4 blocker, potently inhibited CRC growth, epithelial-to-mesenchymal transition progression and metastasis^12^. Togel et al. demonstrated that BRD4 blockage downregulated c-Myc and inhibited CRC cell proliferation, which could be further augmented by targeting WNT or MAPK signaling^49^.

To date more than ten BET inhibitors have advanced to early stage clinical trials for patients with different types of cancer^11,13^. However, BRD4 inhibitors were found to only exert limited antitumor activity in patients^11,13^. One possibility is that BRD4 inhibition induces feedback upregulation of the BRD4 protein, leading to modest anti-proliferative ability and minor apoptotic induction^8,11,13^. Therefore, a new therapeutic approach is urgently needed to target BRD4 and other BET proteins^8,11,13^.

The BRD4 PROTACs have two covalently linked protein-binding domains: one capable of engaging an E3 ubiquitin ligase, and the other binding to BRD4 protein for ubiquitination-mediated degradation^50^. These compounds differ significantly from small-molecular BRD4 inhibitors in their cellular potency, phenotypic effects, pharmacokinetic kinetics and potential toxicity profiles^50^. A1874 is a first-in-class BRD4-targeting MDM2-based PROTAC^14^. Studies have shown that it results in robust and sustained BRD4 degradation^14^. Furthermore, A1874 increases p53 stabilization and protein levels in a dose-dependent manner^14^.

Here, in primary colon cancer cells and established HCT116 cells, A1874 potently inhibited cell viability, growth, proliferation and cell cycle progression, as well as cell migration and invasion. Furthermore, the BRD4-degrading PROTAC induced significant apoptosis activation in primary and established colon cancer cells. At the molecular level, A1874 is able to induce BRD4 protein degradation and the downregulation of BRD-dependent genes (c-Myc, Bcl-2 and cyclin D1) in colon cancer cells.

Although A1874-induced robust and potent BRD4 protein degradation, A1874-induced anti-colon cancer cell activity was not solely dependent on BRD4 degradation. First, A1874 was significantly more potent than other known BRD4/BET inhibitors (JQ1, CPI203, I-BET151) at inducing colon cancer cell apoptosis. Second, restoring BRD4 expression by the BRD4 (Mut) construct only partially inhibited A1874-induced anti-colon cancer cell activity. Third, the novel MDM2-recruiting PROTAC remained cytotoxic in the BRD4-KO colon cancer cells. A1874-induced p53 protein stabilization and oxidative stress in colon cancer cells, two actions that are independent of BRD4 depletion. Conversely, the antioxidant NAC and the p53 inhibitor pifithrin-α attenuated A1874-induced colon cancer cell apoptosis. Therefore A1874 acts via both BRD4-dependent and BRD4-independent (p53 stabilization and ROS production) mechanisms, providing an explanation for its superior anticancer activity against colon cancer cells.

Colon cancer and other CRC are among the third most common type of cancer, accounting for around 10% of all malignancies^3,51^. In 2018, there are 1.09 million new cases and 551,000 CRC deaths (mainly colon cancer). The 5-year survival rate of CRC in the United States is close to 65%^3,51^. Molecularly targeted therapies are the current focus of research for colon cancer^52,53^. Here we report that oral administration of a single dose of A1874 potently inhibits colon cancer xenograft growth in SCID mice. These results demonstrate that this novel compound is a promising therapeutic to treat colon cancer.
